# Early onset, development and histological features of gastric signet-ring cell carcinoma

**DOI:** 10.3389/fonc.2023.1166549

**Published:** 2023-07-06

**Authors:** Yangkun Wang, Yingying Li, Bin Wang, Dongmei Ran, Chaoya Zhu, Ping Li, Bo Jiang, Sunan Wang

**Affiliations:** ^1^ Department of Pathology, Shenzhen Longgang District Fourth People’s Hospital, Shenzhen, China; ^2^ Shenzhen Polytechnic, Shenzhen, China; ^3^ Department of Radiation Therapy, Cancer Center, Shanghai Jiahui International Hospital, Shanghai, China; ^4^ Department of Pathology, Southern University of Science and Technology Hospital, Shenzhen, China; ^5^ Department of Pathology, Third Affiliated Hospital of Zhengzhou University, Shenzhen, China; ^6^ Department of Pathology, Peking University Shenzhen Hospital, Shenzhen, China; ^7^ Department of Pathology, No. 990 Hospital of The People's Liberation Army (PLA) Joint Logistics Support Force, Zhumadian, China

**Keywords:** gastric foveolar epithelial type, histopathology, immunohistochemistry signet-ring cell carcinoma, signet-ring cell differentiation, signet-ring-like heterocysts

## Abstract

**Objective:**

To explore the early onset, development and histological features of gastric signet-ring cell carcinoma (SRCC).

**Methods:**

Three hundred and sixty-two patients with differentiated adenocarcinoma with signet-ring cells were enrolled. Histomorphological and immunohistochemical features and patterns of the specimens were observed in detail.

**Results:**

Infection of the gastric mucosa, especially by *Helicobacter pylori*, can cause massive cell proliferation and transformation in the deep gastric foveola, the isthmus of the gastric gland, and the proliferative zone of the upper neck of the gland. Signet-ring-like heterocysts monoclonally proliferated after the redifferentiation and reproliferation, extending horizontally along the gastric foveola. Gastric foveolar-type SRCC grew infiltratively into the lamina propria of the mucosa and the submucosa, signet-ring cells could differentiate into undifferentiated adenocarcinoma with signet-ring cell differentiation, mucinous adenocarcinoma with signet-ring cell differentiation, gastric adenocarcinoma with signet-ring cell differentiation, and fundus gland adenocarcinoma with signet-ring cell differentiation.

**Conclusion:**

Early SRCC developed from the proliferative zones of the fundus of the gastric foveola and the neck of the gastric gland, growing horizontally along the gastric foveola. It developed into gastric adenocarcinoma with signet-ring cell differentiation after reproliferation and retransformation in the mucosa.

## 1 Introduction

Gastric signet-ring cell carcinoma (SRCC) is a special type of gastric adenocarcinoma with a high malignancy and low diagnosis rate of early SRCC (1–3). However, research in recent years has indicated a link between gastric and intestinal-related immunophenotypes and the biological behavior and prognosis of gastric SRCC (4–6). According to different marker expressions, SRCC can be divided into gastric carcinoma and intestinal carcinoma, with the gastric carcinoma being further divided into small foveolar epithelial (surface mucous epithelium) type, pyloric gland type, and fundus gland type (7, 8). Histologically, the fundus of each gastric foveola that is collected with 3–5 glands is the basic structure unit of the gastric mucosa, and is known as the gastric unit; the structure is of monoclonal origin (9). Cells proliferate actively in the isthmus and the upper neck of the gastric gland. Gastric SRCC originates in the proliferative zone (10). However, this zone contains a variety of poorly differentiated cells with distinct differentiation directions, and the origin of SRCC in the proliferative zone remains unclear (11, 12). It is widely accepted that almost all malignancies experience atypical hyperplasia before onset, with a few transforming directly from normal conditions to malignancies without experiencing atypical hyperplasia of epithelium (13–15). The World Health Organization (WHO) has classified *H. pylori* infection as a carcinogen of gastric carcinoma, and it has become a trend to prevent gastric carcinoma by eliminating *H. pylori* through *H. pylori* detection and treatment (16). A large number of studies have found that *H. pylori* causes changes in the morphology and signal transduction of gastric epithelial cells (17–20). About 10% of gastric cancer (GC) and 1% of colorectal cancer (CRC) are characterized by signet ring cell carcinoma. SRCC is associated with poor prognosis, but the underlying molecular features remain unclear (21). Regardless of the site in which SRCC occurs, it has similar molecular features. Multistage carcinogenesis of SRCC involving genetic and epigenetic aberrations is associated with stage-dependent prognosis (22). There are significant differences in clinicopathological features and prognosis between signet ring cell carcinoma (SRC) and intestinal type gastric cancer (ITGC) (23). In gastric cancer with signet ring cell (SRC) component, there is a good prognosis in stage I. However, the prognosis is poor in stage II/III (24).

The results of our previous research revealed that *H. pylori* selectively adhered to and destroyed the cytoplasm of mucous cells on the surface of the gastric mucosa, causing the ovoid and spherical mucus-containing particles wrapped in limitans in the cytoplasm on the nucleus to disappear, the cytoplasm showed cobweb-like vacuolar degeneration, and the epithelial cells on the surface of the gastric mucosa exfoliated, leading to the proliferation and transformation of the mucous neck cells in the proliferative zone, the formation of papillary epithelioma-like hyperplasia of mucous cells on the surface of the gastric mucosa, the extensive and segmental atrophy of the lamina propria gland of the gastric mucosa, and other changes (21, 22). Studies have shown *H. pylori* infection could cause foveolar-type signet-ring cell carcinoma, and the histopathological features and differential diagnosis of this type of gastric carcinoma have been proposed (23). A total of 362 patients with differentiated adenocarcinoma with signet-ring cells were enrolled in this study. Histomorphological and immunohistochemical features and patterns of the specimens were observed in detail. Our aim was to provide pathological assistance to clinicians in the precise treatment and tracking of stem cell proliferation and transformation in the proliferative zone, enabling them to intervene in the onset and development of gastric SRCC as early as possible.

## 2 Materials and methods

### 2.1 Materials

From May 2020 to May 2022, 362 patients diagnosed with foveal epithelial hyperplasia of gastric mucosa, dysfunction, abnormal proliferation, and transformation by gastroscopic biopsy and who developed differentiated adenocarcinoma with signet-ring cells were enrolled from Shenzhen Longgang District Fourth People’s Hospital, the Third Affiliated Hospital of Zhengzhou University, Peking University Shenzhen Hospital, and the 990th Hospital of the PLA Joint Logistic Support Force. ESD resection or surgical local excision was conducted for 15 patients diagnosed with small signet-ring cell carcinoma-like lesions, foveolar-type signet-ring cell carcinoma, and adenocarcinoma with signet-ring cell differentiation by gastric mucosa biopsy.

### 2.2 Methods

Specimens were fixed with fresh 10% neutral buffered formalin solution (NBF) for 8–48 h within 30 min after detachment *via* biopsy and surgery, with a fixative to tissue volume ratio of 10:1. Materials were collected and sectioned in a standardized manner according to the gastric ESD specimens and early gastric carcinoma specimens (24). H&E, special staining, and immunohistochemistry (IHC) staining were performed.

### 2.3 IHC staining

Using the En Vision two-step method, the tissue sections were deparaffinized, hydrated, and rinsed with distilled water. The sections were then placed in tris-buffered saline (TBS) for 10 minutes. The endogenous peroxidase was blocked for another 5 minutes, and the sections were treated with TBS for 10 minutes. The primary antibodies (CKpan, CK7, CK20, villin, HER2, MUC5AC, ki-67, Hp) were incubated with the sections for 30 minutes at room temperature. The sections were incubated in the En Vision™ after being washed in TBS for 10 min. The sections were washed in TBS for 10 min, and were then added with the secondary antibody, and allowed to react for 10 minutes. After incubating the chromogenic substrate solution for 10 minutes, it was rinsed with distilled water. DAB was used to develop the sections and hematoxylin was used to counterstain them. The known gastric mucosa sections were used as the positive control, while the PBS buffer solution was used as the negative control instead of primary antibodies. All working solutions were purchased from MXB Biotechnologies, and the operation procedures were carried out in strict accordance with the kit instructions.

## 3 Results

### 3.1 Clinical characteristics

Among the 362 patients studied, there were 226 (62.4%) males and 136 (37.7%) females. Among them, there were 142 (39.2%) patients with an onset age ≤ 60 and 220 (60.8%) with an onset age > 60. The relationship between the onset age and gender is shown in **Table 1**.

**Table 1:** Relationship between early onset and development of gastric signet-ring cell carcinoma and the onset age and gender.

Stage	N (%)	Gender	Age
		Male (female)	
Foveal epithelial hyperplasia of gastric mucosa	164 (45.3)	101 (61.6), 63 (38.4)	65 (39.6), 99 (60.4)
Proliferative dysfunction of stem cells in proliferative zone	98 (27.1)	62 (63.2), 36 (36.7)	37 (37.8), 61 (62.2)
Abnormal proliferation and transformation of stem cells in proliferative zone	73 (20.2)	45 (61.4), 28 (38.4)	27 (37.0), 46 (63.0)
Signet-ring-like heterocysts	12 (3.3)	8 (66.7), 4 (33.3)	5 (41.7), 7 (58.3)
Micro signet-ring carcinoid lesion	6 (1.7)	4 (66.6), 2 (33.3)	3 (50.0), 3 (50.0)
Gastric foveolar-type signet-ring cell carcinoma	2 (0.6)	1 (50.0), 1 (50.0)	1 (50.0), 1 (50.0)
Adenocarcinoma with signet-ring cell differentiation	7 (1.9)	5 (71.4), 2 (28.6)	4 (57.1), 3 (42.9)
Undifferentiated adenocarcinoma with signet-ring cell differentiation	3 (0.8)	2 (66.7), 1 (33.3)	2 (66.7), 1 (33.3)
Mucinous adenocarcinoma with signet-ring cell differentiation	2 (0.3)	1 (50.0), 1 (50.0)	1 (50.0), 1 (50.0)
Adenoid adenocarcinoma with signet-ring cell differentiation	1 (0.3)	1 (100.0), 0 (0.0)	1 (100.0), 0 (0.0)
Fundus gland adenocarcinoma with signet-ring cell differentiation	1 (0.3)	1 (100.0), 0 (0.0)	0 (0.0), 1 (100.0)
Total	362	226 (62.4), 136 (37.7)	142 (39.2), 220 (60.8)

### 3.2 Schematic diagram of early onset and development of gastric SRCC

**Figure 1:**
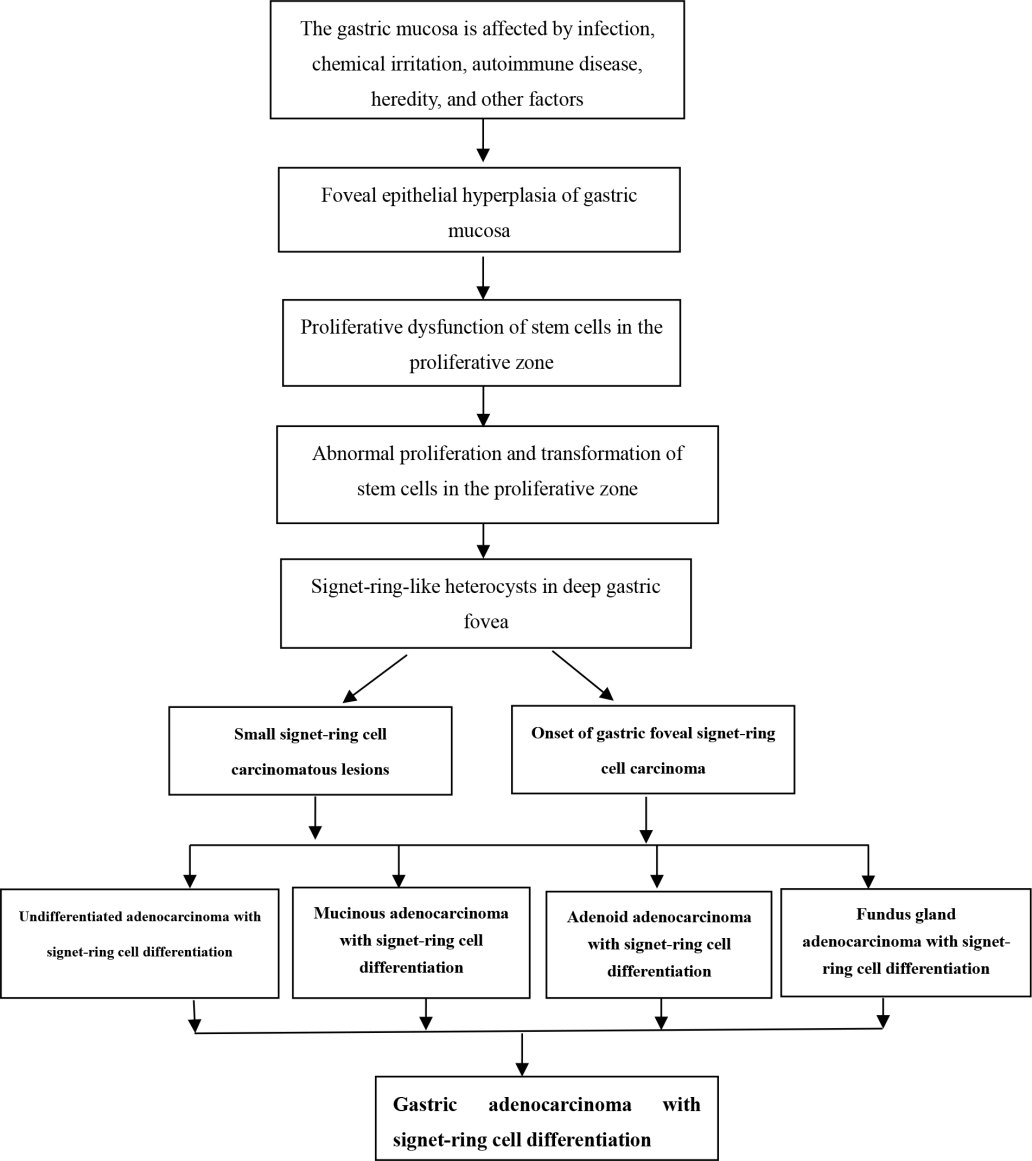
Schematic diagram of early onset and development of gastric signet-ring cell carcinoma.

When the gastric mucosa was affected by infection, chemical irritation, autoimmune disease, heredity, and other factors, it may result in foveal epithelial hyperplasia of gastric mucosa, dysfunction, abnormal proliferation, and transformation, which then developed into differentiated adenocarcinoma with signet-ring cells, as shown in **Figure 1**.

### 3.3 Early histological features of gastric SRCC

**Figure 2:**
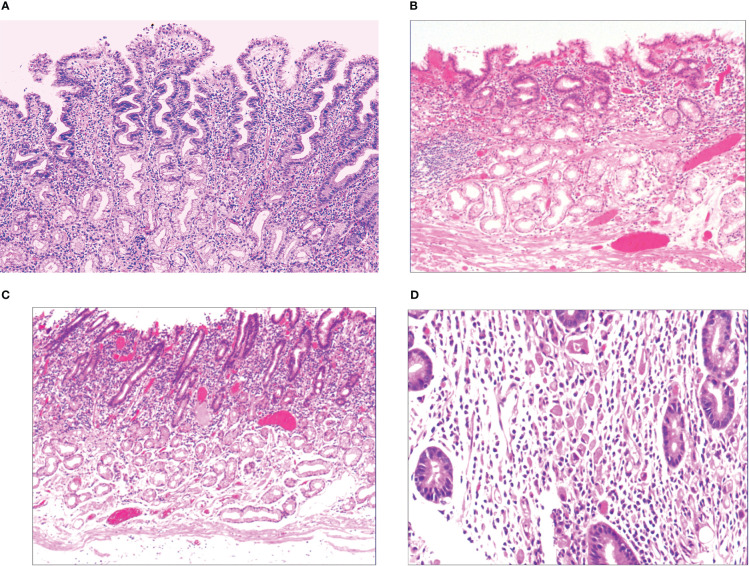
**(A)** Foveal epithelial hyperplasia of gastric mucosa, with height of gastric fovea ≥ 0.05–0.55 mm. The cytoplasm and nucleus of the proliferating surface mucous cells showed a hazy basophilic degeneration, and the nucleus volume increased, and was 1 time the volume of normal nucleus, with a small nucleolus available. HE ×100. **(B)** Proliferative dysfunction of stem cells in the proliferative zone. While there was an insufficient upward migration of the proliferative zone, resulting in a reduction of gastric foveal epithelium, there was an insufficient downward migration of the proliferative zone, resulting in extensive atrophy of the laminar propria glands of the gastric mucosa. HE ×100. **(C)** There was proliferation and transformation of stem cells in the proliferative zone, the nucleus was prolonged, with mild to moderate atypia, the nuclear chromatin increased, with a small to medium-sized nucleolus visible in approximately 20%~30% of the nucleus. HE ×100. **(D)** Signet-ring-like heterocysts were 1–2 times the number of cells on the peripheral columnar epithelium in quantity, and their nucleus showed a crescent shape or irregularly oval shape, forming the signet-ring cell carcinoid cells in cytology, HE ×200.

**Figure 3:**
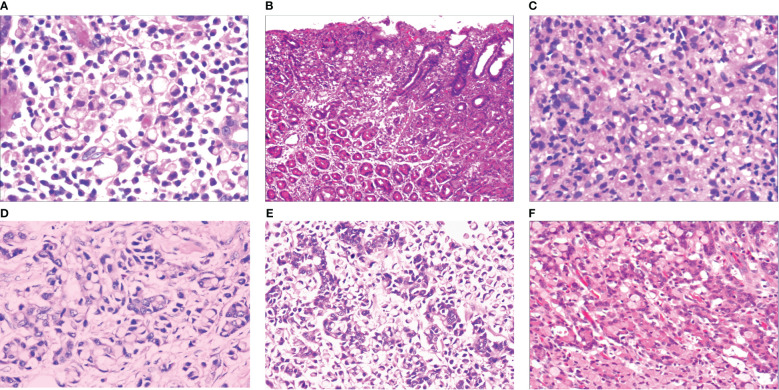
**(A)** Micro signet-ring cell carcinomatous lesion, in which multiple signet-ring cells were close to each other tightly, and were basically friendly toward surrounding normal glands, without mutual aggression. The cytoplasm on the cell was lightly eosinaceous, the envelope area was deeply stained, ringed, and about 70% of the cells were without nucleus. HE ×400. **(B)** Gastric foveolar-type signet-ring cell carcinoma, which also showed horizontal growth in gastric foveal area selectively. It grew slowly and by creeping into the interstitial area of the gastric fovea, and was basically friendly towards surrounding normal glands, without mutual aggression. HE ×100. **(C)** Undifferentiated adenocarcinoma with signet-ring cell differentiation, refers to the tumor formed by a mixture of undifferentiated cancer cells and signet-ring cells. Undifferentiated cancer cells had small volume, irregular small round shape, oval shape, less cytoplasm, a very big proportion between nucleus and cytoplasm, bare nucleus-like, and slightly basophilic staining. Signet-ring carcinoma cells accounted for 20%~80% of the tumor. HE ×200. **(D)** Mucinous adenocarcinoma with signet-ring cell differentiation, consists of signet-ring carcinoma and mucous tissues in terms of tumor histology. Signet-ring carcinoma cells accounted for 20%~80% of the tumor. HE ×200. **(E)** Adenoid adenocarcinoma with signet-ring cell differentiation, consists of signet-ring carcinoma and adenocarcinoma tissues, HE ×200. **(F)** Fundus gland adenocarcinoma with signet-ring cell differentiation, consists of signet-ring carcinoma and fundus gland adenocarcinoma tissues. Fundus gland adenocarcinoma tissues showed irregular tubular and branching structures, with less stroma. HE ×200.

When the gastric mucosa was affected by infection, chemical irritation, autoimmune disease, heredity, and other factors, particularly *H. pylori* infection, it may result in foveal epithelial hyperplasia of the gastric mucosa. The cytoplasm and nucleus of the proliferative surface mucous cell showed hazy basophilic degeneration, the nucleus grew to be about twice the size of normal, and there may be a small nucleoli (**Figure 2A**). It then developed stem cell dysfunction in the proliferative zone (**Figure 2B**). Persistent dysfunction resulted in massive cell proliferation and accumulation in the proliferative zone of the gland’s upper neck, forming a lamellar heterocyst nested structure, known as abnormal proliferation and transformation of stem cells in the proliferative zone (**Figure 2C**). Single or multiple signet-ring cell-like cells were formed because of abnormal proliferation and transformation. This cell was 1-2 times the size of the peripheral columnar epithelium, with its nucleus being crescent or irregularly oval, forming the cytologically SRCC-like cells, which were called signet-ring cell-like heterocysts in the deep gastric foveola (**Figure 2D**). After redifferentiation and reproliferation, signet-ring-like heterocysts developed into classical-type signet-ring cells. The cells were round, with a diameter of 15–30 μm with reddish mucous substances in the cytoplasm; the nucleus was deviated and was signet-ring or crescent-shaped (**Figure 3A**). The foveolar-type signet-ring cell carcinoma was formed when signet-ring-like heterocysts and classical-type signet-ring cells extended horizontally along one third of the opening side of the fundus gland mucosa, with a length of 3–6 mm (**Figure 3B**). When foveolar-type signet-ring cell carcinoma infiltrated into the lamina propria of the mucosa and the submucosa, the signet-ring cell could differentiate into undifferentiated adenocarcinoma with signet-ring cell differentiation (**Figure 3C**), mucinous adenocarcinoma with signet-ring cell differentiation (**Figure 3D**), gastric adenocarcinoma with signet-ring cell differentiation (**Figure 3E**), and fundus gland adenocarcinoma with signet-ring cell differentiation (**Figure 3F**).

### 3.4 IHC staining results

**Figure 4:**
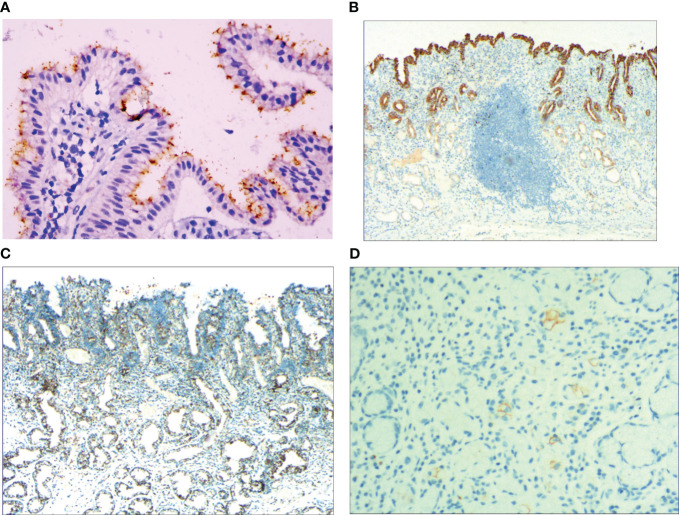
**(A)** Foveal epithelial hyperplasia of gastric mucosa, the helicobacter pylori (*H. pylori*) showed brown expression, by En Vision method, Hp ×400. **(B)** Dysfunction of stem cells in the proliferative zone, reduced MUC5AC positive cells, by En Vision method, ×200. **(C)** Proliferation and transformation of surface epithelial cells, CEA showed positive expression, by En Vision method, ×200. **(D)** Signet-ring-like heterocysts, CK7 showed positive expression, by En Vision method, ×200.

**Figure 5:**
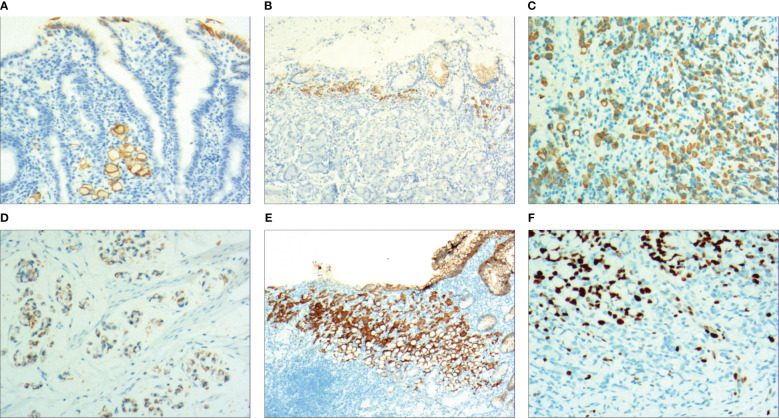
**(A)** Micro signet-ring carcinoid lesion, CK showed positive expression, by En Vision method, ×200. **(B)** Gastric foveolar-type signet-ring cell carcinoma, CK20 showed positive expression, by En Vision method, ×200. **(C)** Undifferentiated adenocarcinoma with signet-ring cell differentiation, CK showed positive expression, by En Vision method, ×200. **(D)** Mucinous adenocarcinoma with signet-ring cell differentiation, HER2 positive 1+, by En Vision method, ×200. **(E)** Adenoid adenocarcinoma with signet-ring cell differentiation, villin signet-ring carcinoma and adenocarcinoma showed positive expression, by En Vision method, ×200. **(F)** Fundus gland adenocarcinoma with signet-ring cell differentiation, ki67 showed positive expression, by En Vision method, Hp ×200.

**Figure 6:**
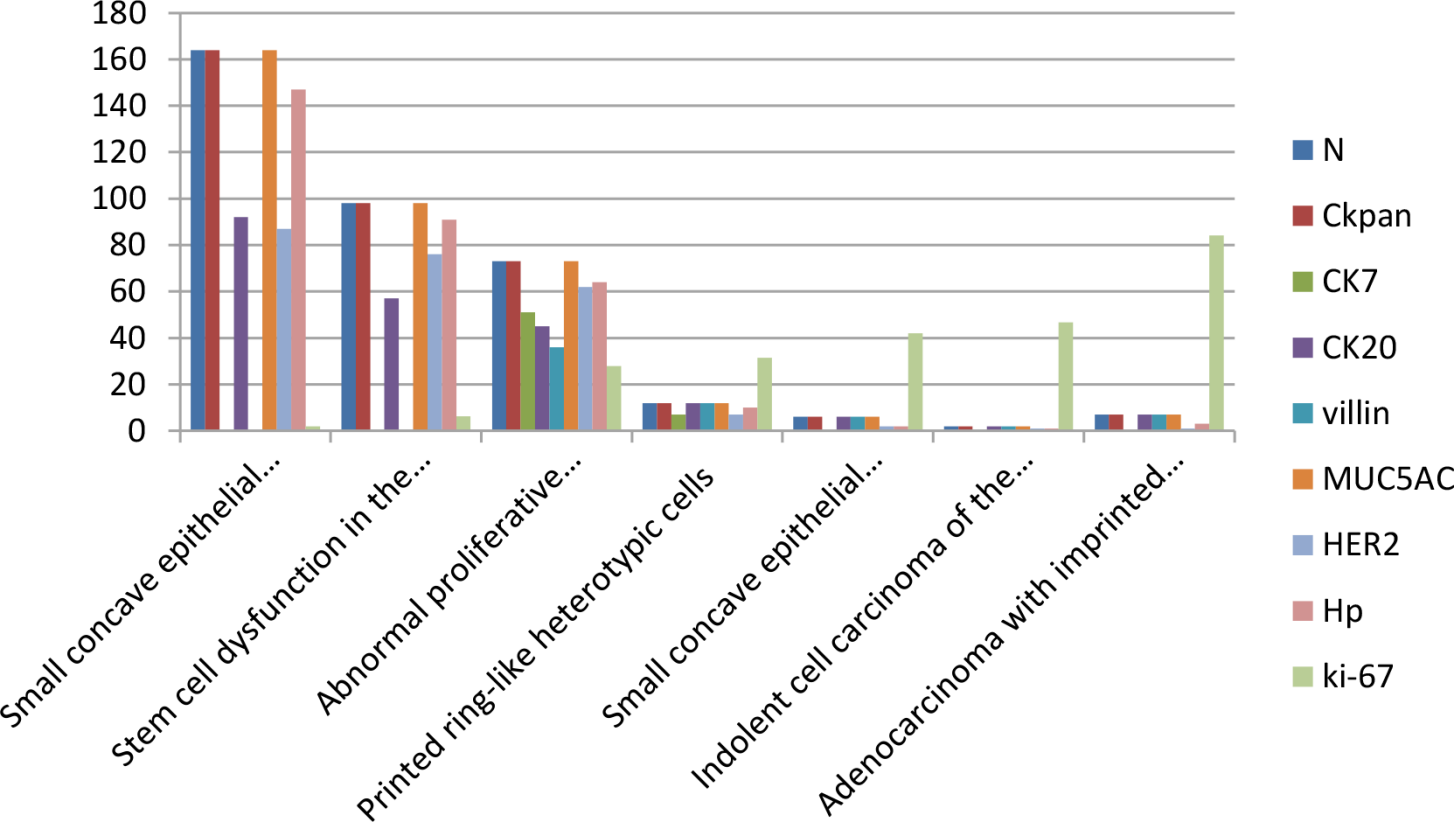
The data of IHC staining results.

With the HP positive cells, *H. pylori* adhered to and selectively destroyed the cytoplasm of the surface mucous cell (**Figure 4A**). MUC5AC was positively expressed on the superficial epithelium and mucous neck gland of normal gastric mucosa, and its expression decreased when stem cells were dysfunctional in the proliferative zone (**Figure 4B**). CEA was positively expressed in cases of abnormal proliferation and transformation of stem cells in the proliferative zone (**Figure 4C**). CK7 was positively expressed in signet-ring-like heterocysts (**Figure 4D**). CK was positively expressed in small signet-ring cell carcinoma-like lesions (**Figure 5A**). CK20 was positively expressed in foveolar-type signet-ring cell carcinoma (**Figure 5B**). In undifferentiated adenocarcinoma with signet-ring cell differentiation, CK expression showed undifferentiated cells and signet-ring cells (**Figure 5C**). In mucinous adenocarcinoma with signet-ring cell differentiation, signet-ring cell HER2 was positive 1+ (**Figure 5D**). In gastric adenocarcinoma with signet-ring cell differentiation, the villin expression showed SRCC and adenocarcinoma (**Figure 5E**). In fundus gland adenocarcinoma with signet-ring cell differentiation, ki67 expression showed SRCC and fundus adenocarcinoma (**Figure 5F**). Details are shown in **Table 2**. The results of immunohistochemical staining are shown in **Table 3** and **Figure 6**.

**Table 2:** Clinical histopathological features of early onset and development of gastric signet-ring cell carcinoma.

Histopathological type	Histopathological features
Foveal epithelial hyperplasia of gastric mucosa	When the gastric mucosa was affected by infection, chemical irritation, autoimmune disease, heredity, and other factors, especially *H. pylori* infection, it resulted in foveal epithelial hyperplasia of the gastric mucosa. Histological findings: the height of gastric fovea was ≥0.05–0.55 mm. The cytoplasm and nucleus of the proliferating surface mucous cells showed a hazy basophilic degeneration, and the nucleus volume was increased, which was 1 time the volume of normal nucleus, with small nucleolus available. Foveal epithelial hyperplasia of gastric mucosa is a reactive hyperplasia, and is also referred to as compensatory hyperplasia. Upon eradication of *H. pylori*, the surface epithelial hyperplasia of gastric mucosa in this stage may be restored, therefore it is also referred to as reversible lesion.
Proliferative dysfunction of stem cells in proliferative zone	The gastric mucosa was continuously affected by infection, chemical irritation, autoimmune disease, heredity, and other factors, resulting in proliferative dysfunction of stem cells in the proliferative zone. While there was an insufficient upward migration of the proliferative zone, resulting in a reduction of gastric foveal epithelium; on the other hand, there was an insufficient downward migration of the proliferative zone, resulting in extensive atrophy of the laminar propria glands of the gastric mucosa. Lastly, it resulted in obstructed upward migration of the stem cells in the zone from top of the fundus gland to deep gastric fovea; due to changes in physiologic regularity or polarity of stem cell proliferation in the zone from top of the fundus gland to deep gastric fovea, a disordered proliferation state was formed.
Abnormal proliferation and transformation of stem cells in the proliferative zone	Continuous dysfunction of stem cells in the proliferative zone resulted in mass proliferation and accumulation of the cells in the proliferative zone of the deep gastric fovea, isthmus of the gastric gland, and superior glandular neck, thus forming a lamellar heteromorphic cell nest-like structure, which is referred to as abnormal proliferation and transformation of stem cells in the proliferative zone. This refers to epithelial cells of the gastric mucosa which are deviated from normal differentiation, and pathologically speaking, refers to the occurrence of atypia features of cell morphology and tissue structure to different degrees. Such proliferated and transformed cells showed a prolonged nucleus, mild to moderate atypia, and increased nuclear chromatin, with a small to medium-sized nucleolus visible in approximately 20%~30% of the nucleus.
Signet-ring-like heterocysts	This refers to the single cell, multiple cells, or lumpy signet-ring-like cells in the proliferative zone of the isthmus of the gastric gland, and superior glandular neck at the time of continuous abnormal proliferation and transformation. It is at a certain distance from the gland within the stroma. Such cells were 1~2 times the number of cells on the peripheral columnar epithelium in quantity, and their nucleus showed a crescent shape or an irregularly oval shape, forming the signet-ring cell carcinoid cells in cytology, therefore, they are referred to as signet-ring-like heterocysts in deep gastric fovea. Such cells were originally found between normal cervical mucus gland cells, and then gradually migrated to outside of the gland, and then entered the re-differentiation and proliferation stage, thus forming an independent monoclonal or neoplastic proliferation.
Micro signet-ring carcinoid lesion	After the re-differentiation and proliferation stage, signet-ring-like heterocysts formed into classical signet-ring cells. These cells were round in shape, with a diameter of 15~30 μm, and had light-red stained mucous substances; the nucleus was deviated and showed a signet-ring or crescent shape. Such signet-ring-like heterocysts and classical signet-ring cells formed proliferative nodes jointly, with the node size ≤ 0.5 mm. These nodes were free of hyperplasia of fibrous connective tissues, extracellular mucus, or glandular structure, and are thus referred to as micro signet-ring carcinoid lesion.
Gastric foveolar-type signet-ring cell carcinoma	The signet-ring-like heterocysts and the classical signet-ring cells made a horizontal expansion within 1/3^rd^ of the opening side of the fundus gland mucosa jointly, at the length of 3~6 mm, therefore, they are referred to as gastric foveolar-type signet-ring cell carcinoma. Gastric foveolar-type signet-ring cell carcinoma was often accompanied by *H. pylori* infection, gastric mucosal erosion, or mucosal ulcer change. Or it formed local mucosal depression due to the thinning of local mucosa.
Adenocarcinoma with signet-ring cell differentiation	
Undifferentiated adenocarcinoma with signet-ring cell differentiation	When gastric foveolar-type signet-ring cell carcinoma makes an invasive growth towards the mucous lamina propria or sub-mucosa, the signet-ring cells can be differentiated into undifferentiated adenocarcinoma with signet-ring cell differentiation. In terms of the tumor histology composition, it is a tumor composed of a mixture of undifferentiated cancer cells and signet-ring cells. Undifferentiated cancer cells are small in volume, and show an irregular small round shape, an oval shape, have less cytoplasm, and involve a very big proportion between the nucleus and cytoplasm, and are bare nucleus-like, and slight basophilic staining. Signet-ring carcinoma has rich cytoplasm, full of mucus, and the nucleus is pressed to one side of the cytoplasm, showing a “signet-ring” shape.
Mucinous adenocarcinoma with signet-ring cell differentiation	In terms of tumor histology composition, it is composed of signet-ring carcinoma and mucous tissues. Signet-ring carcinoma cells account for 20%~80% of the tumor. Signet-ring cancer cells are distributed in the mucous tissues in a single or diffuse way, and separate the mucous and signet-ring carcinoma cells into irregular lumps at different sizes using fibrous connective tissues. As for invasion characteristics of the tumor, it could invade spaces of the surrounding tissues to infiltrate and destroy them.
Adenoid adenocarcinoma with signet-ring cell differentiation	In terms of tumor histology composition, it is made of signet-ring carcinoma and adenocarcinoma tissues. Adenocarcinoma cells are arranged in an adenoid, fascicular, nest-like structure, and are in a staggered distribution with signet-ring cells. Signet-ring carcinoma cells can either be distributed singularly or with multiple ones close to each other, or form an adenoid, fascicular, nest-like structure together with adenocarcinoma tissues.
Fundus gland adenocarcinoma with signet-ring cell differentiation	In terms of tumor histology composition, it is composed of the signet-ring carcinoma and fundus gland adenocarcinoma tissues. Fundus gland adenocarcinoma tissues show irregular tubular and branching structures, with less stroma. In cytology, the glandular epithelial cells are columnar, the cytoplasm is basophilic, the nucleus shows an irregular round or oval shape, the cells are irregularly arranged, with significant atypia, the nucleus volume is approximately more than 2 times than that of normal master cells, and exhibits significantly visible nucleolus in the nucleus. The signet-ring cancer cells are distributed singularly or diffusely in the fundus gland adenocarcinoma tissues.

**Table 3:** The results of immunohistochemical staining.

Staging	N (%)	CKpan	CK7	CK20	villin	MUC5AC	HER2	Hp	ki-67
Small concave epithelial hyperplasia of the gastric mucosa	164 (45.3)	164 (100.0)	0 (0.0)	92 (56.1)	0 (0.0)	164 (100.0)	87 (53.0)	147 (89.62)	2.0%
**S**tem cell dysfunction in the proliferative zone	98 (27.1)	98 (100.0)	0 (0.0)	57 (58.2)	0 (0.0)	98 (100.0)	76 (77.60)	91 (92.9)	6.3%
Abnormal proliferative transformation of stem cells in the proliferative zone	73 (20.2)	73 (100.0)	51 (69.9)	45 (61.4)	36 (49.3)	73 (100.0)	62 (84.9)	64 (87.7)	27.9%
Printed ring-like heterotypic cells	12 (3.3)	12 (100.0)	7 (58.3)	12 (100.0)	12 (100.0)	12 (100.0)	7 (58.30)	10 (83.3)	31.4%
Small concave epithelial hyperplasia of the gastric mucosa	6 (1.7)	6 (100.0)	0 (0.0)	6 (100.0)	6 (100.0)	6 (100.0)	2 (33.3)	2 (66.7)	42.1%
Indolent cell carcinoma of the stomach, small concave type	2 (0.6)	2 (100.0)	0 (0.0)	2 (100.0)	2 (100.0)	2 (100.0)	1 (50.0)	1 (50.0)	46.7%
Adenocarcinoma with imprinted cell differentiation	7 (1.9)	7 (100.0)	0 (0.0)	7 (100.0)	7 (100.0)	7 (100.0)	1 (14.2)	3 (42.9)	84.2%

## 4 Discussion

Gastric cancer is a multifactorial disease (25). Both environmental and genetic factors, such as infection, diet, lifestyle, family history, occupational exposure, and ionizing radiation, can affect its occurrence and development (26). Atrophic gastritis, low acidity, and hypergastrinemia have also been reported in the literature as common risk factors. Especially signet ring cell carcinoma, hypergastrinemia caused by autoimmune chronic atrophic gastritis (27). *H. Pylori* infection leads to gastritis and gastric atrophy, progressively progresses to cancer (28). In summary, gastric cancer occurs when the gastric mucosa is affected by infection, chemical stimulation, autoimmune diseases, genetic factors, and other factors. Gastric cancer is a multi-stage disease process determined by the progressive development of mutations in the expression of various genes and epigenetic changes, which are the cause of the disease. More than half of the world’s population is affected with *H. pylori*, with the majority of people asymptomatic and only about 10% developing peptic ulcer, atrophic gastritis, gastric carcinoma, or MALT lymphoma (24, 29, 30). There is growing evidence that *H. pylori* strains, hosts, and other factors together determine the onset and development of the disease (31). Gastric flora, as an important part of the gastric microecological environment, maintains the balance of the gastric environment through various regulatory approaches. When the structure of the flora changes, the balance is bound to be disrupted, leading to the disease (32). *H. pylori* infection has been identified as a carcinogen of gastric carcinoma by the WHO, and it has become a trend to prevent gastric carcinoma by eliminating *H. pylori* through *H. pylori* detection and treatment (33).

The results of our study revealed that when the gastric mucosa was affected by infection, chemical irritation, autoimmune disease, heredity, and other factors, especially *H. pylori* infection, it resulted in foveal epithelial hyperplasia of gastric mucosa, stem cell dysfunction in the proliferative zone, and abnormal proliferation and transformation of stem cells in the proliferative zone. Subsequently, there were single or multiple signet-ring cell-like cells scattered in the proliferative zones in the isthmus of the gastric gland and the upper neck of the gland. Such cells were 1–2 times the size of the peripheral columnar epithelium, with crescent or irregularly oval nucleus, forming cytologically signet-ring cell-like heterocysts. After redifferentiation and reproliferation, signet-ring-like heterocysts, transformed into small signet-ring cell carcinoma-like lesions. In this study, we presented the characteristics of small signet-ring cell carcinoma-like lesions in the stomach: (1) it was primarily caused by *H. pylori* infection; (2) signet-ring cell-like heterocysts were first formed in the proliferative zones in the isthmus of the gastric gland and the upper neck of the gland; (3) signet-ring-like heterocysts, after redifferentiation and reproliferation, developed into classical-type signet-ring cells; (4) the cells were round, with a diameter of 15–30 μm with reddish mucous substances in the cytoplasm; the nucleus was deviated, and was signet-ring or crescent-shaped. The signet-ring-like heterocysts, along with classical-type signet-ring cells, formed proliferative nodules with a diameter of ≤ 0.5 mm; (5) small signet-ring cell carcinoma-like lesions in the stomach were included into the clinical pathological test report, which was extremely important in guiding clinicians in the precise treatment, tracking of malignancy transformation, and controlling the onset and development of gastric carcinoma. This can provide clinicians with very important biomarker value for early diagnosis of the disease.

One important reason for the low rate of early diagnosis of gastric SRCC is that the pathological mechanism of this gastric carcinoma as well as the histological features of precancerous lesions remain unclear (34–36). According to research, small lesions in the lamina propria of the mucosa can be difficult to diagnose by endoscopy, while the incidence of early SRCC confined to the submucosa is higher as the size is greater. Early SRCC has a much lower incidence of lymph node metastasis than moderately and poorly differentiated adenocarcinoma; when SRCC invades beyond the submucosa, tumor cells spread rapidly and widely.

The results of this study revealed that SRCC occurred when single cells, multiple cells, or lamellar signet-ring cell-like heterocysts first formed in the proliferative zone in the isthmus of the gastric gland and the upper neck of the gland, and signet-ring-like heterocysts, after redifferentiation and reproliferation, developed into small signet-ring cell carcinoma-like lesions or foveolar-type signet-ring cell carcinoma. This stage of carcinoma is the classical early gastric SRCC. It includes classical-type signet-ring cell (the cell is round, with a diameter of 15–30 μm and reddish mucous substances in the cytoplasm; the nucleus is deviated, and is signet-ring or crescent-shaped), and those that we previously reported: juvenile signet-ring cell (the cell is round or irregular, and the cytoplasm is strongly eosinophilic; the nucleus is irregularly round or oval, and deviated, and the ratio of nucleus to cytoplasm is 1:1-3; the nuclear chromatin is basophilic and eosinophilic); hyperproliferative type signet-ring cell (the cell is round or oval, the cytoplasm is uniform and slightly stained with basophilic and eosinophilic mucus; the nucleus is large with a prominent nucleolus, and is deviated); nuclear-free vacuolated signet-ring cell (the cell developed due to the relationship between sections during section preparation; the cell still maintained the cytoplastic outline and had reddish mucous substances in the cytoplasm); degenerative signet-ring cell (the size of the cell increased, and was mostly 20 mm–40 μm, or up to 50 μm; the membranes were incomplete, the nucleus was small and slightly stained, and gray, or it had no nucleus) (23). In this study, when small signet-ring cell carcinoma-like lesions or foveolar-type signet-ring cell carcinoma infiltrated into the lamina propria of the mucosa and the submucosa, signet-ring-like heterocysts and small signet-ring cell carcinoma-like lesions, after reproliferation, differentiated into various types of gastric adenocarcinoma with signet-ring cell differentiation, including undifferentiated carcinoma with signet-ring cell differentiation, mucinous adenocarcinoma with signet-ring cell differentiation, gastric gland adenocarcinoma with signet-ring cell differentiation, and fundus gland adenocarcinoma with signet-ring cell differentiation, with no pure SRCC types being found. Foveolar-type signet-ring cell carcinoma extended horizontally along the one third of the opening side of the fundus gland mucosa and crept into the stroma in the gastric foveola. It was 3–6 mm in length, but transformed into a mixed infiltrative gastric adenocarcinoma while growing infiltratively downward. However, at the time of transformation into mixed invasive gastric adenocarcinoma, quantitative differences in signet-ring cell components occur. Most scholars believe that the percentage of signet ring cells in gastric mixed carcinoma is related to the prognosis, and the number of signet ring cells is considered as an independent predictor (37, 38). Combining the quantitative changes and morphological changes of signet ring cells may offer new potential clinic biomarkers and improve the early clinical diagnosis rate. The evolution process and mechanism of gastric signet ring cell carcinoma into mixed invasive gastric adenocarcinoma need to be further studied by more cases.

## 5 Conclusions

The early onset, development and histological features of gastric SRCC are closely related to *H. pylori* infection. Early SRCC developed from the proliferative zones of the fundus of the gastric foveola and the neck of the gastric gland, growing horizontally along the gastric foveola. It developed into gastric adenocarcinoma with signet-ring cell differentiation after reproliferation and retransformation in the mucosa. However, more cases are needed to further study how *H. pylori* infection leads to the proliferation and transformation of cells in the proliferative zone, and the occurrence of signet-ring cell-like heterocysts, small signet-ring cell carcinoma-like lesions, and gastric foveolar epithelial SRCC.

## Data availability statement

The original contributions presented in the study are included in the article/supplementary material. Further inquiries can be directed to the corresponding author.

## Author contributions

Conception and design of the research: YW, SW. Acquisition of data: DR, CZ, PL, BJ. Analysis and interpretation of the data: YL. Statistical analysis: BW. Obtaining financing: YW. Writing of the manuscript: YW, SW. Critical revision of the manuscript for intellectual content: YW, SW. All authors read and approved the final draft. YW, YL, BW, DR, CZ, PL, BJ, SW. All authors contributed to the article and approved the submitted version.
